# The Haldane Commission and Medical Education in London

**Published:** 1913-08-23

**Authors:** 


### THE HALDANE COMMISSION AND MEDICAL EDUCATION IN LONDON.

### The Established System or the "University Ideal"?

As an epilogue to the International Medical Congress the *Times* of August 14 and the two following days contained three interesting and trenchant articles by Sir Henry Morris on the "Training of Doctors." Sir Henry, as an ex-president of the College of Surgeons, late senior surgeon to the Middlesex Hospital, a graduate in arts and medicine and a former examiner in surgery of the University of London, is well able to speak with experience and authority. He takes Lord Haldane's Report on University Education in London as a chopping-block and he subjects its findings to the most ruthless handling. The time for such criticism is most opportune, for it synchronises with the announcement of the appointment of a Departmental Committee by the Board of Education to report "as to the steps by which effect shall be given to the scheme of the Report of the Royal Commission" and to recommend "the specific arrangements and provisions which may be immediately adopted for that purpose and as the basis of the necessary legislation." This rather unusual sequel to a Royal Commission appears to indicate an intention to rush the new scheme for the University into operation as rapidly as the exigencies of legislation may permit. Meanwhile we are reminded that the attempt of the Commission to transfer the University headquarters to a site in Bloomsbury, without consulting its governing body, the Senate, has proved a singularly ill-starred adventure. The *Times* of August 5 justly observed that "it is to the rather masterful methods adopted by Lord Haldane in furtherance of the Bedford (Bloomsbury) Scheme that much of the delay must undoubtedly be attributed; it was obviously essential that the Senate of the University should co-operate in any scheme, and as the governing body it had the first claim to be consulted. Steps were, however, taken by Lord Haldane to acquire the Bedford Site without the cognisance of the Senate, and it is natural that strong protests should have been registered against this attempt to decide the question over their heads."

If half of what Sir Henry Morris tells us in regard to the medical portion of Lord Haldane's Scheme is to be accepted, the case for further and fuller consideration before any fresh "masterful methods" are resorted to to enforce a revolution on the London medical schools is abundantly clear. The Commissioners, who, by the way, did not count a medical man or a graduate of London University amongst them, appear to have swallowed whole a scheme propounded to them by an American gentleman, Mr. Abraham Flexner. In pursuit of the "ideal" sketched by the Commission every medical school would form an integral part of a University; two or three "hospital units" of an American or German pattern would replace the existing independent medical schools in London. Such sacrifice is, we are assured, required in the interests of "research" and the exigencies of a "University atmosphere." Dr. Starling appears to have adopted the Flexner formula as an afterthought, though Sir Henry Morris questions alike his "taste" and his "assertions." The present system of medical education in London is loftily condemned as too utilitarian, its exponents fail to exert any "seminal influence in the way of ideas upon those that come under their influence," and must accordingly be wiped out as inefficient and obsolete. To such superior minds it is doubtful if one person could be found in London fit to be a University professor of surgery, though preadventure "there are in London one or two young men fit to be University professors of medicine."

The ideal of the Commissioners is to make the Medical Faculty uniform with the Science and Arts Faculties; its professors to be paid to devote their whole time to research in, or the teaching of, their subject; to controlling the clinical laboratories and those who work in them; while private practice is, of course, to be deprecated, if not forbidden. Sir Henry Morris believes that but for the "God-send" of this Mr. Flexner's intervention the Commission, like previous Commissions, would have left the clinical teaching, of which the London schools are justly proud, untouched and uncriticised. He foresees that, if the Commission's scheme is forced upon the University, the independence of the medical schools and their attached hospitals would be imperilled and the present Faculty of Medicine in the University would disappear. Moreover, unless all the medical schools were transformed on the "hospital unit" plan into University "constituent colleges" a double system of graduation would obtain, one set of students being examined mainly by their own teachers—"a most undesirable and defective method"—or else treated as "externals." Sir Henry fears that "these graduates would go into practice trained, it may be very well theoretically, but absolutely unfit for the responsibilities of their profession." The whole scheme is conceived not in the interests of the students, nor of the patients, but is a wanton sacrifice of an established system of proved efficiency on the altar of a fantastic "University ideal."

Sir Henry Morris foresees difficulties with the King Edward's Hospital Fund and the Hospital Sunday Fund in the not improbable event of the University being driven to contribute to its school from hospital revenue. "There would," he says, "be an outcry against any assistance being given to a hospital largely used for research, and in the laboratories of which vivisection would be freely resorted to,'' and he fears, too, that the hospitals "might be impaired from too much interference with the comfort and quiet of the patients under University control and research." Sir Henry traces the growth and justifies the existence of the London medical schools and inquires, "Why should we graft on to our British methods a system which has been as pernicious in its origin as it is unpractical in character and results," and which may be described, in Hegel's words, as "a conception without existence in fact." "Why," he asks, in conclusion, "should an attempt be made to thrust on the University of London such a series of recommendations—a set of cast-iron professors in a miniature faculty, figuratively feeding a few students in a sort of doll's-house 'constituent college' with fertilising ideas, forming a section of an ideally perfect University with German foundations and a Maryland crest, and flying the Banner of the Great Ideal? Why should it?" The question is answered in the asking. Unsigned articles have appeared recently in the *Quarterly* and in the *Westminster Gazette* lauding the Report of the Commission as a "masterly treatise," and one of its members has written to the *Times* again pushing the claims of the "ideal site" for the "ideal University" in the purlieus of Bloomsbury.

To this a rejoinder appeared on August 14 by Mr. T. Hynes, who recalls the questionable origin of the Commission, its unfortunate *personnel*, and its unconcealed hostility to that open-examination system of the University of London, which, nevertheless, made the reputation of its degrees during the latter half of last century. He also recalls the fact that the Bloomsbury Site was at one time all but "foisted upon the University" for a sum nearly three times the value placed upon it by the County Council's valuer.

Whatever may be the merits or demerits of other portions of Lord Haldane's Report, we are satisfied that the case presented by Sir Henry Morris merits the most serious consideration, and any attempt precipitately to force the Commission's Scheme upon the London hospitals and medical schools must raise formidable opposition. The whole future of our voluntary hospitals is at the present time uncertain and the occasion for not a little apprehension on the part of their best friends. Gratuitously to complicate the question by attempting to Americanise or Germanise our London medical schools cannot fail to provoke an embittered and quite unnecessary controversy. No system on the Continent nor in the United States can show more thorough grounding in the subsidiary sciences, more painstaking and practical clinical instruction, or more genuine regard for the welfare of the patients. For sanity of judgment, just application of science to practice, and high ethical ideals our London teaching is unsurpassed. It is no mere regard for "the wisdom of our ancestors" nor in the spirit of the *laudator temporis acti* that we refuse to join in Lord Haldane's demand for the surrender of such substance with a view to replace it by the shadowy substitute recommended by Mr. Abraham Flexner.

